# ANGPTL8 reverses established adriamycin cardiomyopathy by stimulating adult cardiac progenitor cells

**DOI:** 10.18632/oncotarget.13061

**Published:** 2016-11-03

**Authors:** Shuyuan Chen, Jiaxi Chen, Xing-Li Meng, Jin-Song Shen, Jing Huang, Pintong Huang, Zhaoxia Pu, Nathan H. McNeill, Paul A. Grayburn

**Affiliations:** ^1^ Baylor Research Institute, Dallas, TX, USA; ^2^ the University of Texas Southwestern Medical Center at Dallas, Medical School, Dallas, TX, , USA; ^3^ Department of Ultrasonography, The 2nd Affiliated Hospital of Zhejiang University College of Medicine, Hangzhou, Zhejiang Province, China; ^4^ Department of Internal Medicine, Division of Cardiology, Baylor Heart and Vascular Institute, Baylor University Medical Center, Dallas, Texas, USA

**Keywords:** Adriamycin cardiomyopathy, Angiopoietin-like protein 8, Ultrasound targeted microbubble destruction (UTMD), Progenitor cells, Paired immunoglobulin like-receptor B (PirB)

## Abstract

Established adriamycin cardiomyopathy is a lethal disease. When congestive heart failure develops, mortality is approximately 50% in a year. It has been known that ANGPTLs has various functions in lipid metabolism, inflammation, cancer cell invasion, hematopoietic stem activity and diabetes. We hypothesized that ANGPTL8 is capable of maintaining heart function by stimulating adult cardiac progenitor cells to initiate myocardial regeneration. We employed UTMD to deliver piggybac transposon plasmids with the human *ANGPTL8* gene to the liver of rats with adriamycin cardiomyopathy. After *ANGPTL8* gene liver delivery, overexpression of transgenic human *ANGPTL8* was found in rat liver cells and blood. UTMD- *ANGPTL8* gene therapy restored LV mass, fractional shortening index, and LV posterior wall diameter to nearly normal. Our results also showed that *ANGPTL8* reversed established ADM cardiomyopathy. This was associated with activation of ISL-1 positive cardiac progenitor cells in the epicardium. A time-course experiment shown that ISL-1 cardiac progenitor cells proliferated and formed a niche in the epicardial layer and then migrated into sub-epicardium. The observed myocardial regeneration accompanying reversal of adriamycin cardiomyopathy was associated with upregulation of PirB expression on the cell membrane of cardiac muscle cells or progenitor cells stimulated by *ANGPTL8*.

## INTRODUCTION

Adriamycin (ADM) is one of the most widely prescribed and effective cytotoxic drugs used in oncology. However, the utility of adriamycin is limited by cumulative, dose- related, progressive myocardial damage that may lead to congestive heart failure (CHF). Thus, patients who may benefit from continued administration of the drug must withdraw from adriamycin therapy and switch to an alternative agent, which may be less effective [1]. An estimated cumulative percentage of 5% of adriamycin induced CHF patients at a cumulative dose of 400 mg/m^2^, 26% of patients at 550 mg/m^2^, and 48% of patients at 700mg/m^2^. Although adriamycin cardiomyopathy accounts for only a small number of heart failure cases compared to ischemic cardiomyopathy or idiopathic dilated cardiomyopathy, it is a lethal disease. When congestive heart failure develops, mortality is approximately 50% in a year. Moreover, it often occurs in relatively young patients with curable malignancies such as breast cancer. Adriamycin is well known for its cardiac toxicity during chemotherapy for cancer. The cardiotoxicity is due to free radical formation and lipid peroxidation, which results in changes in lysosomes, sarcolemma's, mitochondria, and sarcoplasmic reticulum. These changes induce calcium overload, activation of hydrolytic enzyme, and reduction in energy production. Reduction of cardiac function was a result of the loss of structural integrity. Because adriamycin cardiotoxicity is dose dependent, it has been used to predictably induce heart failure in different animal species considered as a decent animal model with heart failure [2-6]. It will be very crucial to study how to prevent or cure adriamycin cardiomyopathy for these cancer patients. Traditional medical therapy for CHF is directed toward relief of symptoms and blockade of neurohormonal activation. In patients with advanced stages of CHF, heart transplant or left ventricular assist devices may be appropriate, but are expensive and have limited availability. The ideal goal for CHF therapy is myocardial regeneration. It is currently known that cardiac myocytes are capable of regeneration, via various mechanisms, including self-replication of pre-existing adult cardiac muscle cells [7-8], differentiation of resident cardiac progenitor cells [9-10], dedifferentiation and proliferation of adult cardiac muscle cells [11-13] and transdifferentiation of fibroblast cells into cardiac muscle cells [14-15]. However, the magnitude of myocardial regeneration triggered by such mechanisms is small and it remains unclear whether myocardial regeneration in heart failure is sufficient to reverse established cardiomyopathy. Multiple clinical trials of stem cell therapy have been attempted but without clear improvement in cardiac function [16]. Part of this problem may be the fact that patients with advanced HF often have lack of blood supply to the heart and extensive scar tissue due to ischemic cardiomyopathy or long-standing non-ischemic cardiomyopathy with extensive fibrosis. The LIM homeodomain transcription factor (ISL-1 or Islet1) is a biomarker of cardiac progenitors or neural stem cells that give rise to the right ventricle, atria and outflow tract during cardiac development [17]. These cells can differentiate to the different cell types that form an adult heart (cardiomyocytes, smooth muscle cells, and endothelial cells) [18-19], However ISL-1 positive progenitor cells are usually quiescent during adult heart, being present in parasympathetic neurons, smooth muscle cells, a few cardiomyocytes within the proximal aorta and pulmonary artery and more abundantly in the sinoatrial node. The epicardium contains epicardium-derived cells (EPDCs), which undergo epithelial-to-mesenchymal transition (EMT) and differentiate into all main cell lineages of the heart [20]. However some evidence shows that adult EMCs primarily undergo fibrogenic EMT upon cardiac stress, such as hypertension or infarction, to generate myofibroblast-like cells [21]. These cells can contribute to the development of cardiac disease, such as fibrosis, potentially leading to impaired cardiac performance and arrhythmias, including sudden death. It is very crucial to find out the molecular and cellular aspects of these EMCs as well as associated factors, and their implication in EMT and cardiac fibrosis in order to prevent or treat certain heart diseases.

Angiopoietin-like proteins are a family of 8 member proteins that are structurally similar to the angiopoietins, all containing an amino-terminal coiled-coil domain, a linker region, and a carboxy-terminal fibrinogen-like domain except *ANGPTL8*. *ANGPTLs* have various functions in lipid metabolism, inflammation, cancer cell invasion, hematopoietic stem activity. *ANGPTL8* (also known as *C19orf80*) is a 596 bp cDNA with a short sequence in the N-terminus that is highly enriched in the liver and adipocytes [22-27]. Yi et al [28-29] found that a hepatic overexpression of *ANGPTL8* after a hydrodynamic injection of non-viral sleeping beauty transposon plasmids into the tail vein of adult mice had several effects: it stimulated the proliferation of pancreatic beta cells, increased insulin secretion, improved glucose tolerance and expanded the mass of the beta cell islets. We have confirmed the pancreatic beta cell proliferation induced by *ANGPTL8,* but it was not able to reverse diabetes in rats with STZ- induced diabetes [30] or obese rhesus monkey with naturally occurring diabetes [data not shown]. To our knowledge, the effects of *ANGPTL8* on myocardial regeneration in heart failure have not been reported. Our group developed a novel approach to gene therapy by targeting the delivery of non-viral DNA to the organs using ultrasound-targeted microbubble destruction (UTMD) [31-37]. In this study, we employed UTMD to deliver the *ANGPTL8* gene to the liver of rats with established cardiomyopathy induced by adriamycin. We found that hepatic overexpression of *ANGPTL8* can reverse established cardiomyopathy.

## RESULTS

### UTMD targeting of human ANGPTL8 gene to liver of rats

**Figure 1:**
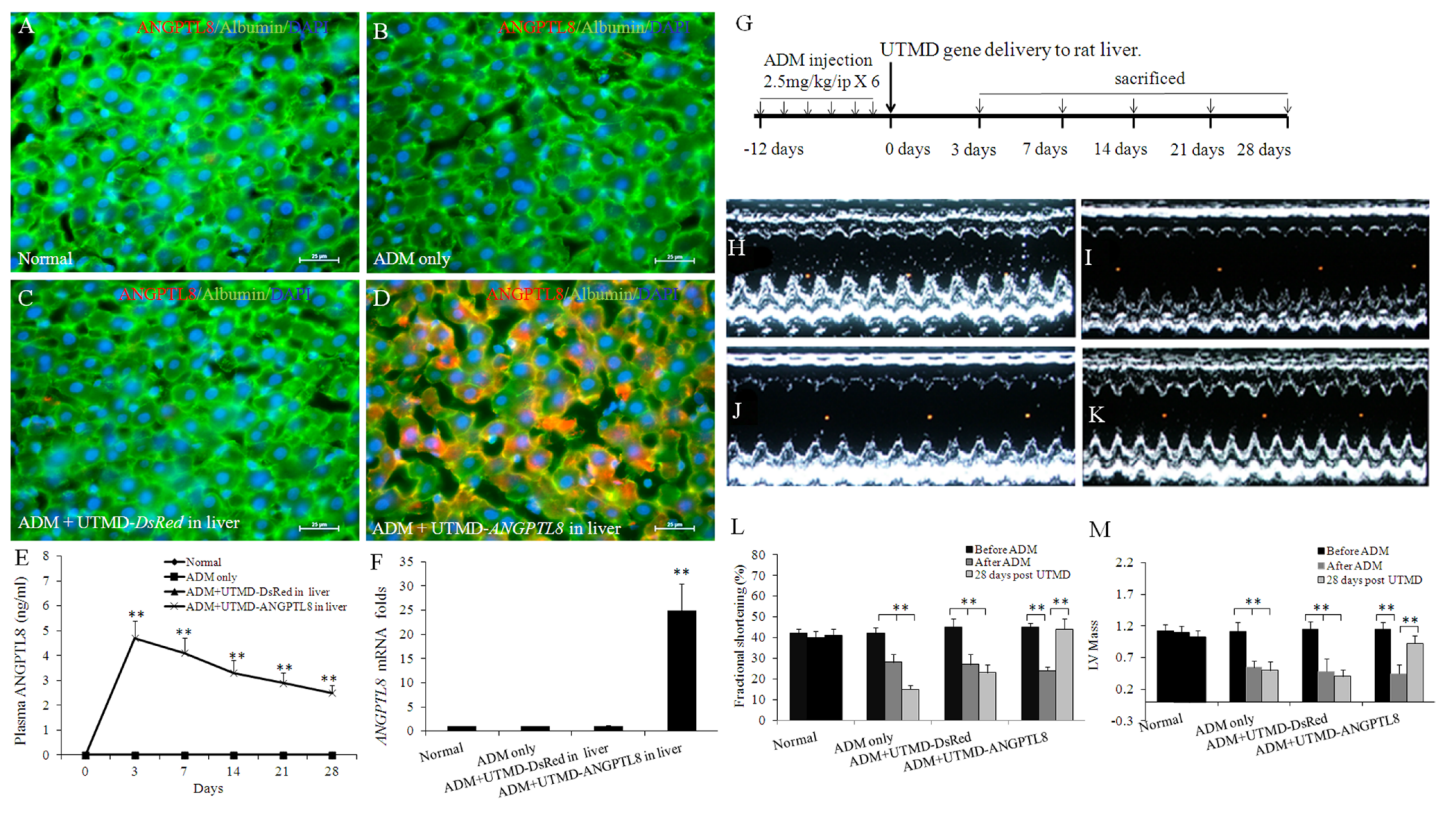
UTMD delivered ANGPTL8 gene to liver (**A**-**D**) triple staining with ANGPTL8/Albumin/DAPI for normal rat liver (**A**), liver of ADM only rat (B), ADM plus UTMD-pXL-BASII-CI-DsRed/hyPB targeted to rat liver (**C**), ADM plus UTMD- pXL-BASII-CI-*ANGPTL8*/pCI-*hyPB* targeted to liver (**D**). Scale bar is 25 μm. (**E**) a graphic of fasting plasma human *ANGPTL8* levels. In normal, ADM control and ADM plus UTMD-*DsRed* groups, human *ANGPTL8* was not detectable. Values are presented as mean ± SEM. *n* = 6 per group; **P<0.001 *vs* control groups. (**F**) a graphic of RT-qPCR for human *ANGPTL8* mRNA, housekeeping gene is *actin* gene. Values are presented as mean ± SEM. *n* = 6 per group; **P<0.001 *vs* control groups. (**G**) Schematic indicating time-course experiment. (**H**-**K**) Results of echocardiographic measurement of cardiac structure and function. M-mode images at 2D parasternal short axis of the left ventricle, (**H**) normal rat heart, (**I**) ADM control rat heart, (J) ADM plus UTMD-DsRed rat heart, (**K**) ADM plus UTMD-ANGPTL8 rat heart. (**L**) a graphic of fractional shortening, (**M**) a graphic of LV mass, Values are presented as mean±SEM. *n* = 6 per group; **P<0.001 *vs* after ADM.

Figure 1 shows that human ANGPTL8 signal was detected in liver cells after UTMD-pXL-BASII-CI- ANGPTL8/pCI-hyPB delivered to the liver (Figure 1D) but was not detected in normal rat liver, nor in the controls treated with ADM only and ADM plus UTMD-DsRed reporter gene (Figure 1A-1C). Human ANGPTL8 signal was detected in the cytoplasm of liver cells of rats from 3 days to 4 weeks after delivery of the ANGPTL8 gene to liver by UTMD. Figure 1E shows fasting plasma levels of human ANGPTL8 in rats. In the normal and DsRed reporter gene control rat groups, no human ANGPTL8 was detected in fasting plasma. However, in the treatment groups, human ANGPTL8 was detected in fasting plasma from day 1 to day 28 after UTMD. We further evaluated human ANGPTL8 mRNA levels using qRT-PCR. The results (Figure 1F) showed that human ANGPTL8 mRNA levels after UTMD-ANGPTL8 gene delivery were 25± 8 fold greater, respectively, than for the normal, ADM only and ADM plus UTMD-DsRed control groups (p<0.001). Western blotting was employed to detect human ANGPTL8 from liver protein extracts, and the results showed that the human ANGPTL8 signal existed in the liver protein extracts after UTMD-ANGPTL8 gene delivery (data not shown).

### Reversal of established adriamycin cardiomyopathy after UTMD-ANGPTL8 gene therapy

We decided to evaluate if there is any possible pharmacological effects of ANGPTL8 on rat hearts with established adriamycin cardiomyopathy. We employed echocardiography to evaluate heart structure and function in all groups. Figure 1H-1K demonstrated M-mode images derived from 2D parasternal short axis views of the left ventricle showing decreased LV fractional shortening and LV mass in adriamycin cardiomyopathy with restoration to normal values by UTMD-ANGPTL8 gene therapy (Figure 1L and 1M).

**Figure 2:**
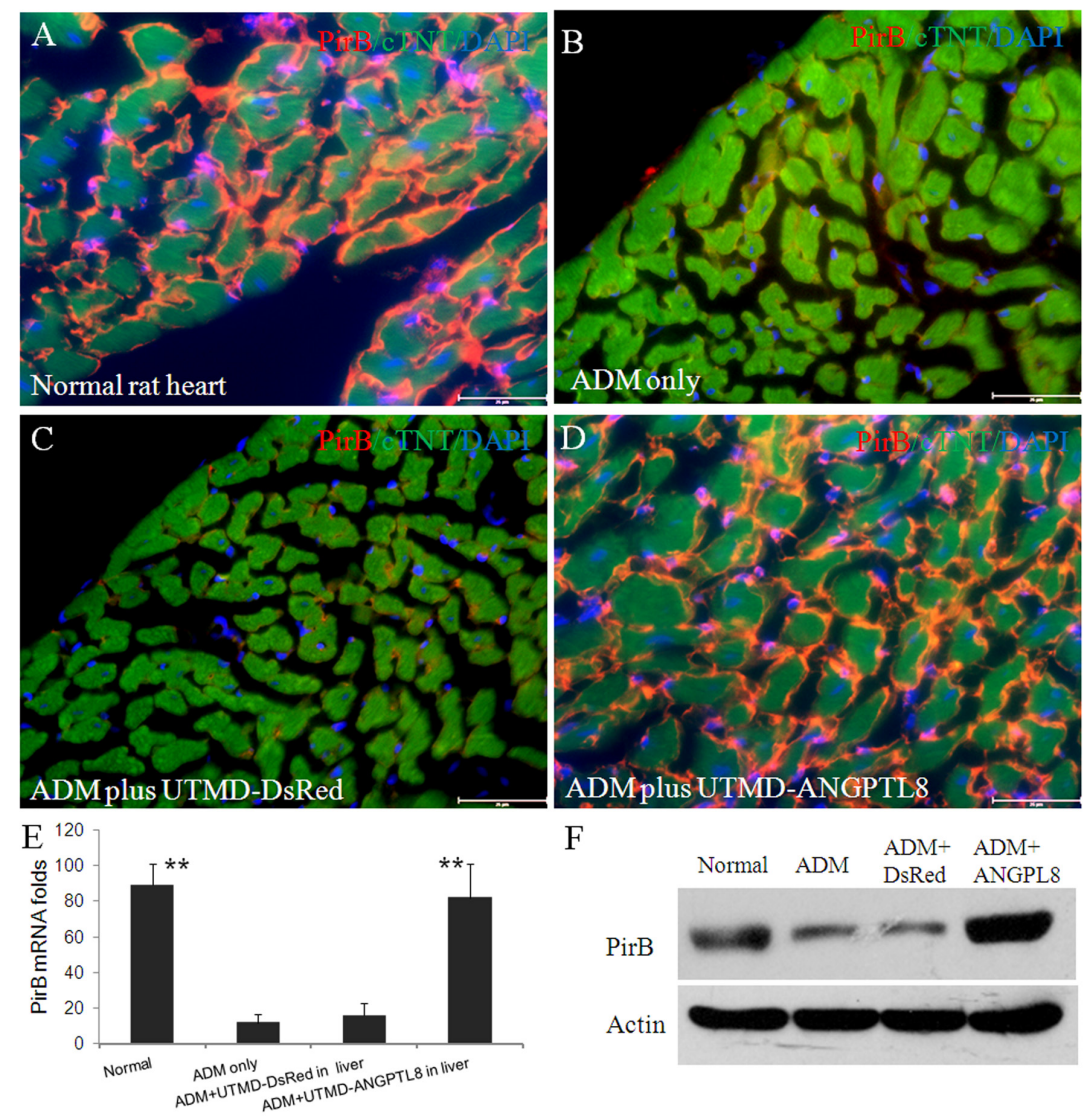
Upper panel (A-D): Paired immunoglobulin-like receptor B (PirB) distributed on membrane of cardiac muscle cells of adult rat (**A**) Normal rat heart; (**B**) ADM only control heart; (**C**) ADM plus UTMD-DsRed heart; (**D**) ADM plus UTMD-ANGPTL8 heart; Scale bar is 25 μm. Red color for PirB signal; green for cardiac troponin T signal; blue for nucleus identity. (**E**) a graphic of RT-qPCR for PirB mRNA, housekeeping gene is *actin* gene. Values are presented as mean ± SEM. *n* = 6 per group; **P<0.001 *vs* ADM only and ADM plus DsRed groups. (**F**) Western blots detecting PirB from protein extracts of rat heart tissue, Actin is a marker of cytoplasm proteins.

### ANGPTL8 significantly upregulated PirB expression on membrane of cardiac muscle cells

**Figure 3:**
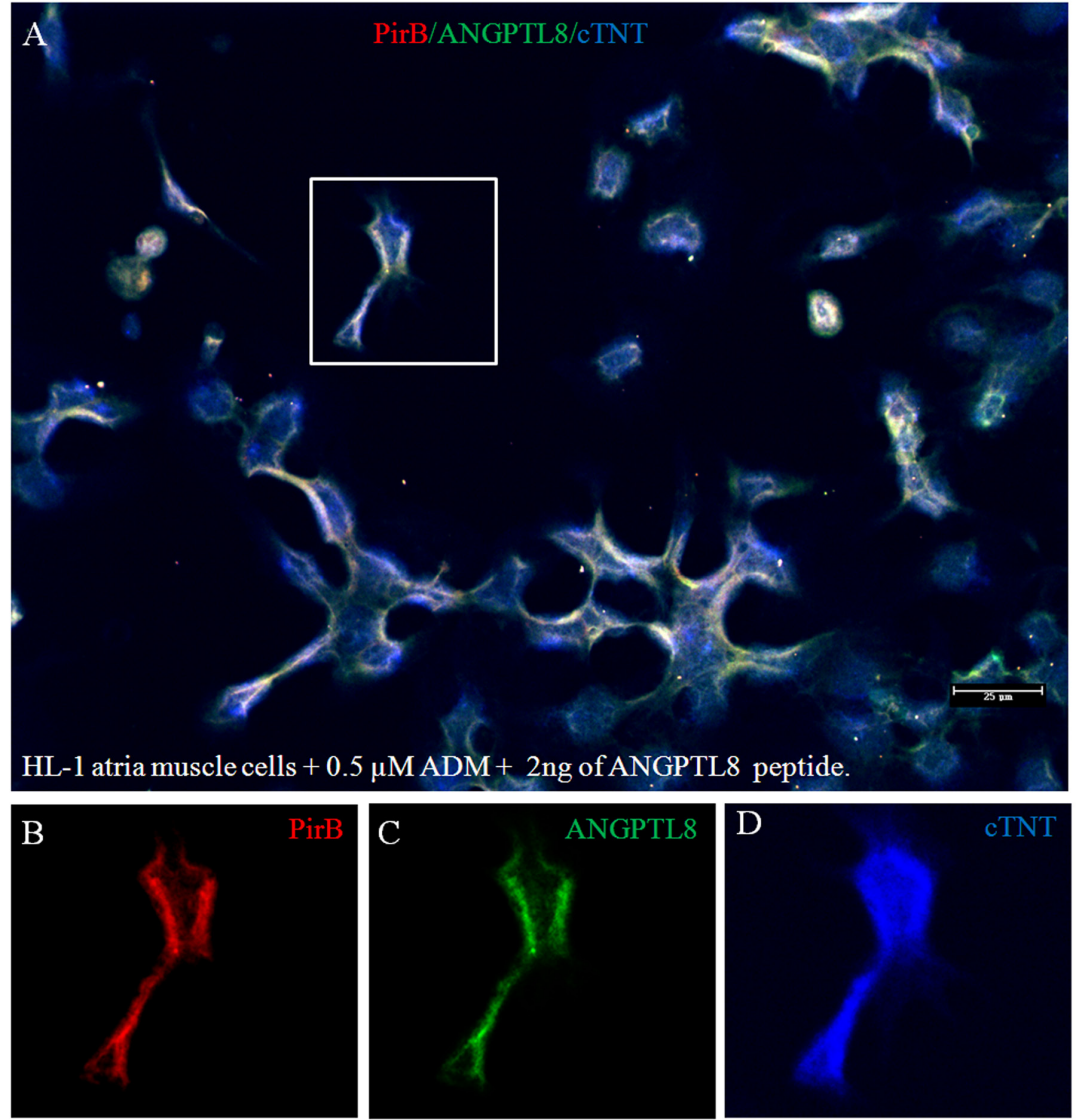
The culture atria muscle cell line (HL-1) treated with 0.5 μM ADM plus 2 ng of ANGPTL8 peptide Scale bar is 25 μm. Red color for PirB signal; green for ANGPTL8 signal; blue for cardiac troponin T.

Zheng, et al [38] reported that Paired immunoglobulin-like receptor B (PirB) in rodents or LILRB2 in human is a specific receptor of *ANGPTLs* in membrane of hematopoietic stem cells. *ANGPTLs* stimulate expansion of hematopoietic stem cells *ex vivo* by activating PirB. It also is an inhibitory receptor in neurons that inhibited regeneration of adult neuron cells [39]. However, there are no reports of PirB in adult cardiac muscle cells. Figure 2 shows robust presence of PirB on the surface of cTNT positive cardiac muscle cells (Figure 2A). However, under established cardiomyopathy induced by ADM, PirB was rarely seen (Figure 2B-2C), after reversal of ADM cardiomyopathy by *ANGPTL8* gene liver delivery, PirB was again seen on the surface of membrane of cardiac muscle cells (Figure 2D). We quantified PirB mRNA levels using qRT-PCR. The results (Figure 2E) showed that PirB mRNA levels are 89±16, 12±4, 16±6, and 82±24 folds respectively in normal rat heart, ADM only control heart, ADM plus UTMD-DsRed heart, and ADM plus UTMD-ANGPTL8 heart groups. The results of western blotting shows that PirB existed in all the heart protein extracts, was significantly deceased in ADM treated heart samples, and recovered after UTMD-ANGPTL8 gene delivery (Figure 2F). We decided to use cultured mouse HL-1 atrial cardiomyocyte cells to confirm what we found *in vivo* experiment, Figure 3A shown that PirB clearly localized on the membrane of HL-1 atrial cardiomyocyte cells as a membrane receptor, and exogenously ANGPTL8 peptide are binding with PirB on the membrane of HL-1 atrial cardiomyocyte cells (Figure 3B-3D).

### ANGPTL8 significantly activated ISL-1 positive cardiac progenitor cells in epicardial layer

**Figure 4:**
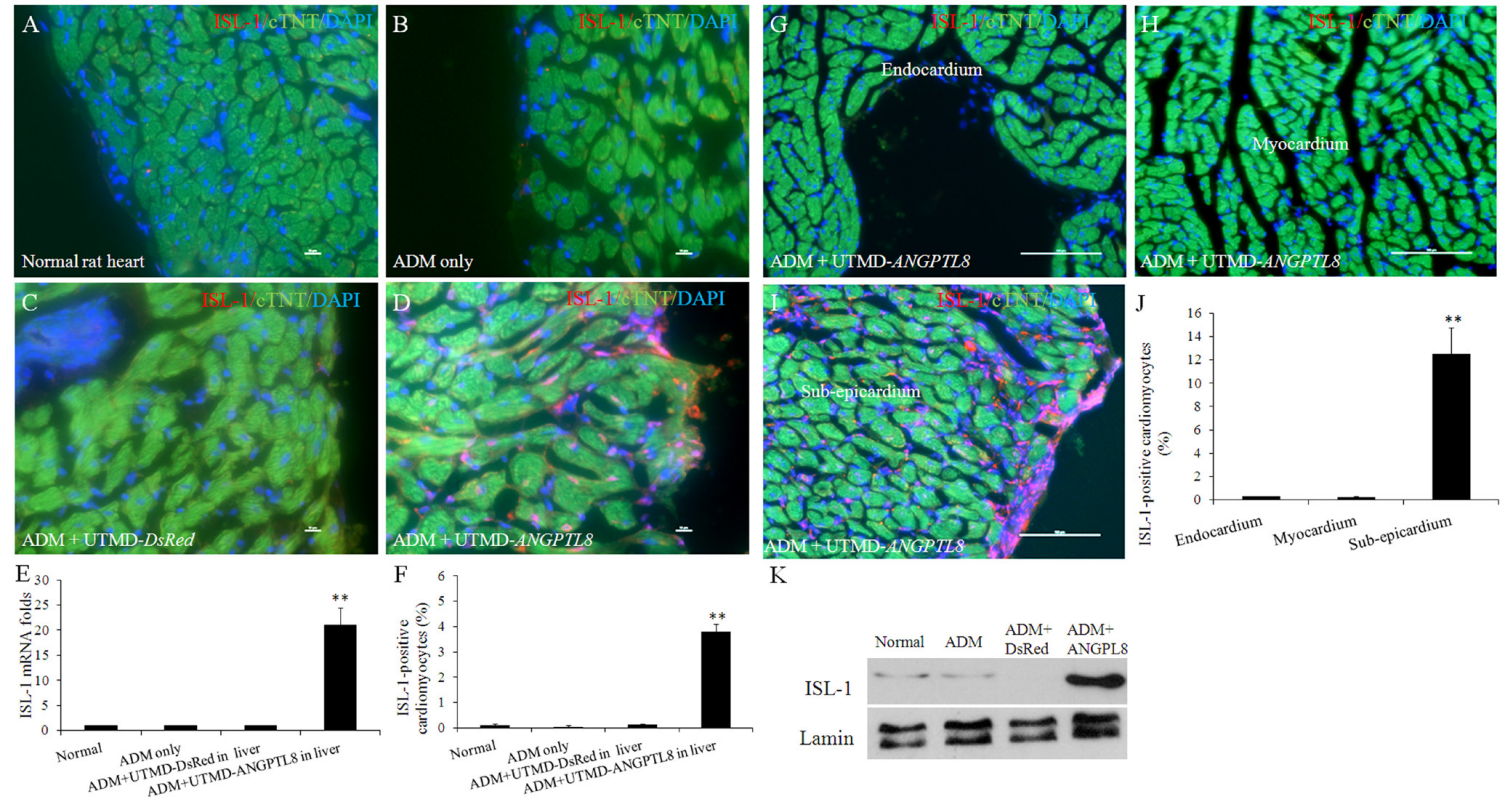
The activation of ISL-1 (an early cardiac muscle differentiation marker) in epicardium and sub-epicardial layer after ADM plus UTMD-ANGPTL8 (**A**) normal rat heart, (**B**) ADM control rat heart, (**C**) ADM plus UTMD-DsRed, (**D**) ADM plus UTMD-ANGPTL8. Scale bar is 10 μm. (**E**) a graphic of qRT-PCR for ISL-1 mRNA, housekeeping gene is *actin* gene. Values are presented as mean ± SEM. *n* = 6 per group; **P<0.001 *vs* control groups. (**F**) a graphic for percentage of ISL-1-positive cardiomyctes cells. Values are presented as mean ± SEM. *n*=6 per group; **p<0.001 vs control groups. The distribution of ISL-1-positive adult cardiac progenitor cells after ADM plus UTMD-ANGPTL8. (**G**) Endocardium, (**H**) Myocardium, (**I**) Sub-epicardium, at hearts of ADM plus UTMD-ANGPTL8 group. Scale bar is 100 μm. (**J**) a graphic for percentage of ISL-1-positive cardiomyoctes cells. Values are presented as mean ± SEM. *n*=6 per group; **p<0.001 vs endocardium and myocardium. (**K**) Western blots detecting ISL-1 from nuclear protein extracts of rat heart tissue, Lamin is a marker of nuclear protein.

Figure 4D shown that ISL-1 positive signals were seen in nucleus of cardiac muscle cells 28 days after ADM plus UTMD- *ANGPTL8* but not in other groups (Figure 4A-4C). The percentage of ISL-1 positive cardiac muscle cells was 3.8±1.2% in ADM plus UTMD- *ANGPTL8* group (p<0.001 vs controlling groups) (Figure 4F). qRT-PCR shown that mRNA level of ISL-1 is 21-fold higher in ADM plus UTMD- *ANGPTL8* compared to controls (p<0.001) (Figure 4E). The results of western blotting shown that ISL-1 signal was scant in nuclear protein extracts of normal heart, ADM only and ADM plus UTMD-DsRed heart samples, but significantly increased in after UTMD-ANGPTL8 gene delivery(Figure 4K). Finally, the location of ISL-1 positive cells was confined to the epicardium and sub-epicardium (Figure 4I) and not seen in endocardium and myocardium (Figure 4G-4H and 4J) (P<0.001).

**Figure 5:**
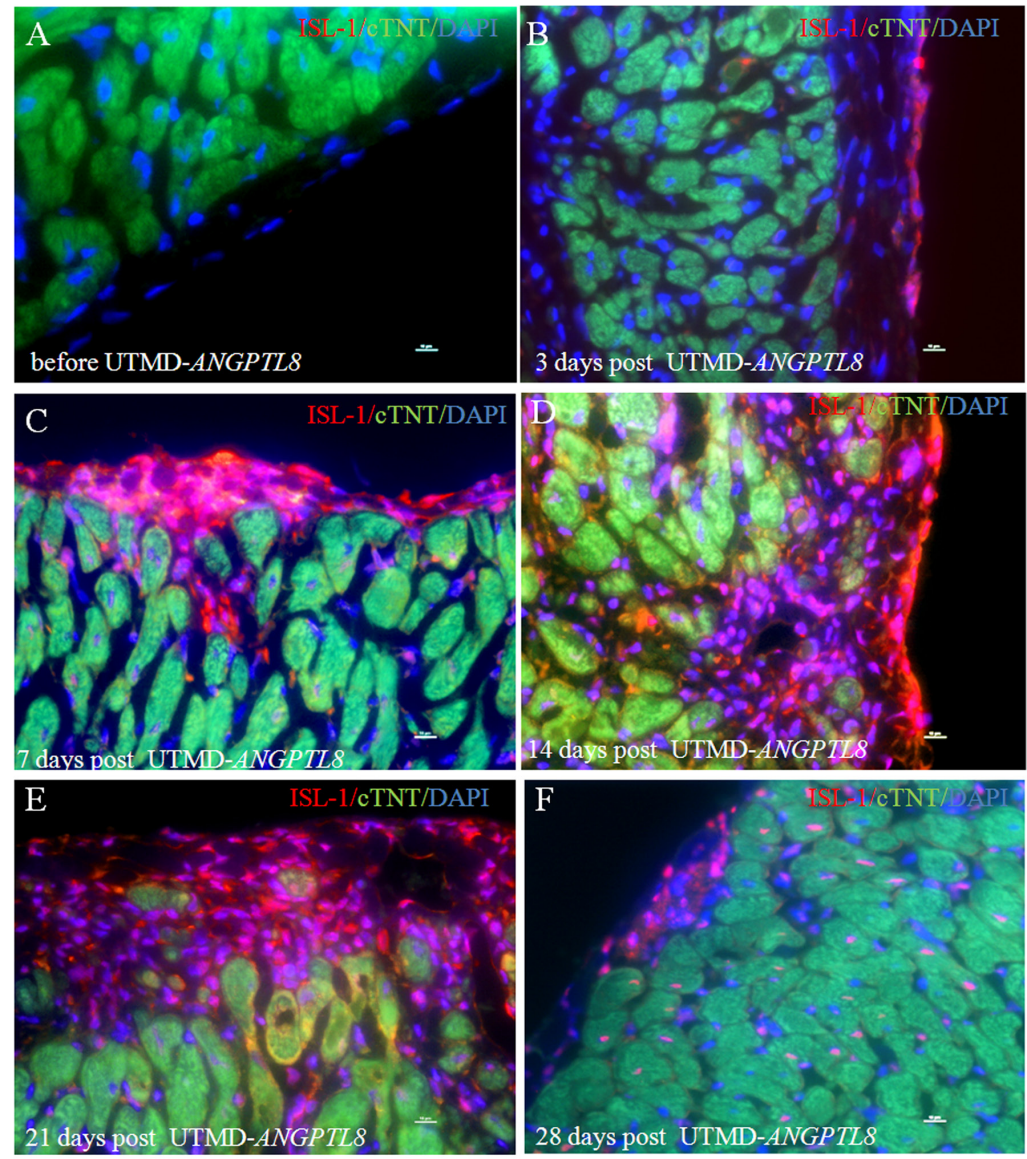
A time-course experiment show that ANGPTL8 induced epicardial layer cells (**A**) into ISL-1-positive cardiac progenitor cells (**B**) and formed niches of cardiac progenitor cells in epicardium (**C**) proliferated and migrated into myocardium layer (**D**-**E**) and differentiate into new cardiac muscle cells (**F**). Scale bar is 10 μm.

We decided further to investigate the effect of *ANGPTL8* on ISL-1 positive cardiac progenitor cells with a time-course experiment. Figure 5 shows that no ISL-1 signal was seen in epicardial layer cells before UTMD- *ANGPTL8* gene delivery (Figure 5A), but was observed 3 days post UTMD- *ANGPTL8* treatment (Figure 5B). ISL-1 positive cells appeared to cluster into epicardial niches of cardiac progenitor cells 7 days post UTMD- *ANGPTL8* gene delivery (Figure 5C), further proliferating and migrating into sub-epicardial myocardium (Figure 5D-5E) and finally differentiating into new cardiac muscle cells (Figure 5F).

### Regenerating cardiac muscle cells are in proliferation

**Figure 6:**
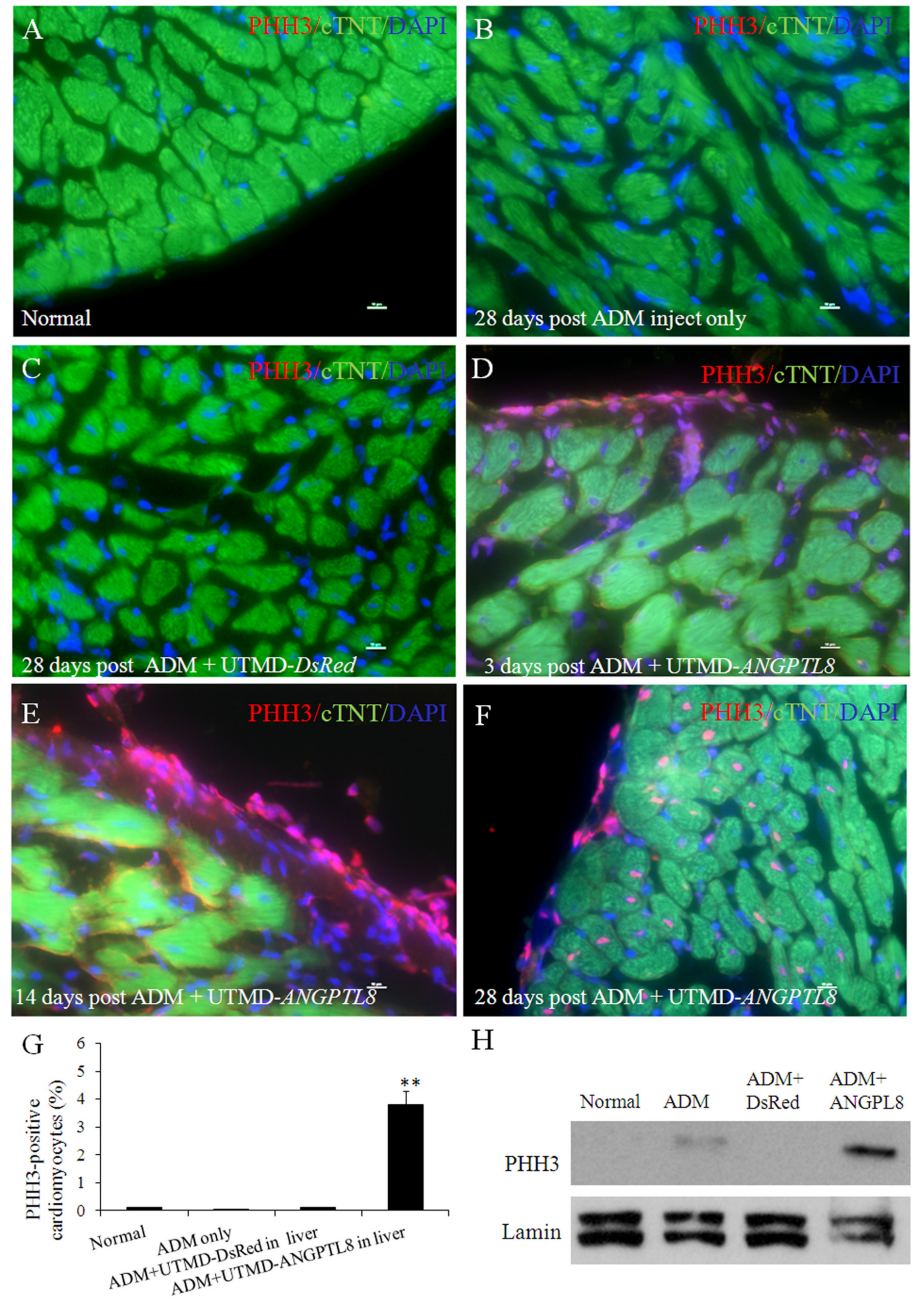
Phospho-histone H3 (PHH3) staining shown some regenerating cardiac muscle cells are in proliferation (**A**) normal rat heart, (**B**) 28 days post ADM inject , (**C**) 28 days post ADM plus UTMD-DsRed, (**D**) 3 days post ADM plus UTMD-ANGPTL8. (**E**) 14 days post ADM plus UTMD-ANGPTL8. (**F**) 28 days post ADM plus UTMD-ANGPTL8. Scale bar is 10 μm, (**G**) Percentage of PHH3-positive cardiomyctes cells. Values are presented as mean ± SEM. *n*=6 per group; **p<0.001 vs control groups. (**H**) Western blots detecting PHH3 from nuclear protein extracts of rat heart tissue, Lamin is a marker of nuclear protein.

We used a mitotic marker (anti-phospho-histone H3 (Ser10) (PHH3)) to demonstrate if regenerating cardiac muscle cells were proliferating. We calculated the percentage of PHH3 positive cardiomyocytes by counting stained nuclei (pink color) from 1000 cTnT positive cardiomyocytes cells in the sub-epicardium using serial sections through each rat heart (n=6 each group). Figure 6 shows that PHH3 signal was observed within the nuclear positive cells by confocal microscopy in ADM plus UTMD- *ANGPTL8* groups. The percentage of PHH3 positive sub-epicardial muscle cells in the rats treated with UTMD- *ANGPTL8* gene therapy 28 days post-UTMD (Figure 6F) was 3.8±0.60% (p<0.001 vs controls). Figure 6D-6E shows evidence of cell proliferation (PHH3 expression) confined to the epicardial layer. The western blotting results showed that PHH3 signal was not seen in the adult heart nuclear protein extracts except in the ADM plus UTMD-ANGPTL8 gene delivery hearts (Figure 6H).

### ANGPTL8 protein therapy also reversed established ADM cardiomyopathy

We wonder if exogenous *ANGPTL8* protein delivery has the same effects as its gene liver delivery. We set three different dosage of *ANGPTL8* protein for intramuscular injection daily for 14 days; Supplemental Figure 1A shows fasting plasma levels of human *ANGPTL8* protein in rats. No human *ANGPTL8* was detected in fasting plasma in ADM plus saline injection, in the three different dosages groups. However, human *ANGPTL8* was detected in fasting plasma from day 3, 7, 10 and 14 during daily injection of *ANGPTL8* protein for consistently 14 days. Supplementary Figure 1B-C demonstrated M-mode images derived from 2D parasternal short-axis views of the left ventricle, compared with normal rat heart, ADM plus saline injection control rat heart, ADM plus *ANGPTL8*-5μg/kg rat heart, ADM plus *ANGPTL8*-20μg/kg rat heart and ADM plus *ANGPTL8*-40μg/kg rat heart. There was decreased LV fractional shortening and LV mass in adriamycin cardiomyopathy with some degree of restoration by *ANGPTL8* protein therapy. Supplementary Figure 2 shows that ISL-1 positive cells in epicardial layer were activated by *ANGPTL8* protein injection with a dose dependent manner after 14 days treatment. Supplementary Figure 3 displayed that some epicardial cells and sub-epicardial cardiac muscle cells are in proliferation after *ANGPTL8* protein therapy.

## DISCUSSION

Balmer, et al [40] reported that cardiac progenitor cells in epicardial layer of adult heart are a resource for myocardial regeneration and they are able to differentiate into cardiac muscle cells, coronary artery muscle cells and vascular endothelial cells. They appear to originate from migration of hematopoietic stem cells from bone marrow. Under some pathological states, these cells may differentiate into fibroblasts, forming scar tissue in heart. It will be a crucial to study how to drive these cardiac progenitor cells to differentiate into muscle cells but not fibroblasts. There is some evidence that *ANGPTL1-7* expands hematopoietic stem cells *ex vivo* and stimulates the formation of niches of hematopoietic cells in bone marrow. Our study demonstrates that *ANGPTL8* can stimulate adult myocardial regeneration by proliferation of cardiac progenitor cells located at epicardium. Time-course experiments suggest that *ANGPTL8* drove the differentiation of cardiac progenitor/stem cells into ISL-1 positive cardiac progenitor cells which formed niches and migrated into sub-epicardial myocardium. It still is not clear what the relationship is between WT1 positive progenitor cells and ISL-1 cardiac progenitor cells in epicardium. The exact molecular mechanism by which *ANGPTL8* activated these quiescent adult cardiac progenitor cells in adult hearts with established ADM cardiomyopathy is also not known.

Zheng, et al [38, 41-43] showed that the human immune inhibitory receptor leukocyte immunoglobulin-like receptor B2 (LILRB2) and its mouse ortholog paired immunoglobulin-like receptor (PirB) are receptors for several angiopoietin-like proteins. LILRB2 and PirB are expressed on human and mouse hematopoietic stem cells, respectively, and the binding of *ANGPTLs* to these receptors supported ex vivo expansion of hematopoietic stem cells. In mouse transplantation acute myeloid leukemia models, a deficiency in intracellular signaling of PirB resulted in increased differentiation of leukemia cells, revealing that PirB supports leukemia development. Their study indicated an unexpected functional significance of classical immune inhibitory receptors in maintenance of normal adult stem cells and in support of cancer development. Supplemental Figure 4 show that PirB exists on the surface membrane of ISL-1 positive cardiac progenitor cells. Our study suggests that *ANGPTL8* expands cardiac progenitor cells in epicardium by the activation of PirB signal pathway. Although some data indicates that this inhibitory receptor signal is related to the Notch1 control pathway [44-46], further studies are needed to decipher the role of PirB in adult cardiac muscle cells under physiological or pathological states. Nevertheless, our finding that *ANGPTL8* reverses established ADM induced cardiomyopathy offers hope that pathways exist for myocardial regeneration in adult animals. In conclusion, *ANGPTL8* has previously unrecognized effects that result reversal of established cardiomyopathy by activation of PirB receptor on the cell membrane of resident adult cardiac progenitor cells. Further evaluation of the mechanism of the effect of ANGPTL8 on cardiac PirB receptors is needed.

## MATERIALS AND METHODS

### Animal protocols

Animal studies were performed according to National Institutes of Health (NIH) recommendations and approved by our institutional animal research committee. Adult male Sprague–Dawley rats were purchased from Harlan Laboratories (Indianapolis, IN, USA).

The protocol was planned to test the hypothesis that delivery of ANGPTL8 by UTMD could reverse established adriamycin (ADM) cardiomyopathy defined as a fractional shortening < 30% by echocardiography after injection of ADM [47] at total dose of 15 mg/kg/ip, 2.5mg/kg/ip 6 times over 2 weeks. Roughly 70% of animals developed an established cardiomyopathy after 6 doses of ADM. Only the animals with a fractional shortening <30% were selected into the protocols.

### Protocol-1 for ANGTPL8 gene therapy

There were 120 rats divided into three control groups of 30 rats each and a treatment group of 30 rats: (1) normal control rats; (2) ADM injection only; (3) ADM plus UTMD with a DsRed reporter gene (pXL-BASII-CI-DsRed/pCI-hyPB); (4) ADM plus UTMD with ANGPTL8 (pXL-BASII-CI-ANGPTL8/pCI-hyPB). UTMD day was set as day 0, all rats were euthanized at 3 days, 7 days, 14 days, 21 days and 28 days after UTMD. The thymidine analog, 5-bromo-2-deoxyuridine (BrdU) (100mg/kg) was injected intraperitoneally 6 hours prior to euthanizing.

Male Sprague–Dawley rats (230–270g) were anesthetized with intraperitoneal ketamine (60 mg/kg) and xylazine (5 mg/kg), and a polyethylene tube (PE 50, Becton Dickinson, Franklin Lakes, TN, USA) was inserted into the right internal jugular vein by cut-down. Piggybac transposon donor plasmids and helper plasmids ratio (pXL-BASII-CI-ANGPTL8/pCI-hyPB) was 5:1. Microbubble or control solutions (0.5 ml diluted with 0.5 ml phosphate-buffered solution (PBS)) were infused over 1 min 30 seconds via pump (Genie, Kent Scientific, Torrington, CT). During the infusion, ultrasound was directed to the liver using a commercially available ultrasound transducer (S3, Sonos 5500, Philips Ultrasound, Bothell, WA). Ultrasound was then applied in ultraharmonic mode (transmit 1.3 MHz/receive 3.6 MHz) at a mechanical index of 1.2. Four bursts of ultrasound were triggered to every fourth end-systole by electrocardiogram using a delay of 45–70 ms after the peak of the R wave. These settings have shown to be optimal for plasmid delivery by UTMD using this instrument [48]. Bubble destruction was visually apparent in all rats. After UTMD, the jugular vein was tied off, the skin closed, and the animals allowed to recover. All of rats were euthanized using an overdose of sodium pentobarbital (120 mg/kg).

### Protocol-2 for ANGTPL8 protein therapy

15 rats divided into (1) normal control rats; (2) ADM plus saline injection rats; (3) ADM plus ANGPTL8 protein at dose of 5μg/kg/day intramuscular injection (into the right quadriceps) for 14 days; (4) ADM plus ANGPTL8 protein at dose of 20μg/kg/day intramuscular injection for 14 days; (5) ADM plus ANGPTL8 protein at dose of 40μg/kg/day intramuscular injection for 14 days. All rats were euthanized at 14 days after starting ANGTPL8 protein injection. The thymidine analog, 5-bromo-2-deoxyuridine (BrdU) (100mg/kg) was injected intraperitoneally 6 hours prior to euthanasia. Human recombinant protein ANGTPL8 protein was purchased from Phoenix Pharmaceuticals (Cat # 056-61, Burlingame, CA, USA).

### Manufacture of plasmid-containing lipid-stabilized microbubbles

Lipid-stabilized microbubbles were made as previously described [48]. Briefly, 250 mg of 1,2-dipalmitoyl-sn-glycero-3-phosphocholine, 50 mg of 1,2-dipalmitoyl-sn-glycero-3-phosphoethanolamine and 10% glucose were mixed with PBS and boiled in water until fully dissolved. Next 2 mg of plasmid DNA was mixed with 0.5 ml of ethyl alcohol and centrifuged at 10,000 g for 5 min. The supernatant fraction was removed, and the DNA pellet was placed in an incubator at 37°C for 5 min to remove any remaining ethyl alcohol. The DNA was then added to 50 μl of Lipofectamine 2000 (1 mg/ml; Invitrogen, Carlsbad, CA, USA) and mixed for 20 min. This mixture was added to 250 μl of liposome solution, 5 μl of 10% albumin and 50 μl of glycerol in 1.5 ml vials, and placed on ice. The headspace of the vials was filled with perfluoropropane gas, and the vials were then shaken for 30 seconds at 4°C. The mean diameter and concentration of the microbubbles were 1.9 ±0.2 μm and 5.2 ± 0.3 × 10^9^ bubbles per ml, respectively. The concentration of plasmid carried by the microbubbles was 250±10 μg/ml.

### Plasmid constructs

Human ANGPTL8 cDNA with Sal1/Not1 cutting sites (Integrated DNA Technologies, Coralville, IA, USA) was subcloned into PiggyBac transposon plasmids (pXL-BSII donor plasmid) provided by Dr. Fraser at the University of Notre Dame (Notre Dame, IN, USA) [49], and hyperactive piggyBac transposase helper plasmid was provided by Dr. Bradley at Wellcome Trust Sanger Institute (Cambridge, UK) [50]. Cloning, isolation and purification of the plasmids were performed by standard procedures, and the PCR products were sequenced to confirm that no artefactual mutations were present.

### Immunohistochemistry

Tissue samples were fixed in 10% formalin for 24 hours and transferred into 70% alcohol for paraffin embedding and 4% paraformaldehyde and 20% sucrose overnight at 4 °C for frozen sections. Cryostat sections 5-8 μm in thickness were further fixed with acetone (-20°C) for 5 min and quenched for 5-20 min with 10 mM glycine in PBS. Sections were then rinsed in PBS 3 times, and permeabilized with 0.5% Triton X-100 in PBS for 15 min. The slides that needed further nuclear protein retrieval were subjected to boiling citrate buffer solution with tween 20 at pH 6.0 for 5 minutes. Sections were blocked with Aquablock solution (EastCoast Bio, North Berwick, ME) at room temperature for 1hr and washed with PBS 1 time. The primary antibodies rat anti-PirB, 1: 200 dilution ( BD biosciences, San Jose, CA ) , rabbit anti-Notch1, 1:200, rabbit anti- FoxO1, 1:250, and rabbit anti-ISL-1, 1: 500 dilution, and mouse anti-cardiac troponin T, 1: 250 dilution, rabbit anti-PHH3, 1:200 dilution, rabbit anti-human ANGPTL8 at 1:100 dilution, rabbit anti-albumin, 1:250 dilution (Abcam Inc, Cambridge, MA), mouse anti-human ANGPTL8 1:200 dilution (Phoenix Pharmaceuticals, Burlingame, CA, USA), were added and incubated for 2 hrs at RT or overnight at 4 °C. After washing with PBS three times for 5 min, the secondary antibody (Sigma, St; Louis, MO) anti-mouse lgG conjugated with FITC; anti-rabbit IgG-conjugated with Texas Red, or anti-donkey lgG conjugated with Cy5) (1:250 dilution in block solution) were added and incubated for 1 hr at RT. Sections were rinsed with PBS for 10 min, 3 times, and incubated with Dapi (Invitrogen, Carlsbad, CA), 1: 5000 dilution for 5 min and washed 3 times with PBST, then mounted. A confocal microscope was used to take pictures.

### Culture of HL-1 Atria Muscle Cells line

The HL-1 cell line was a generous gift from Dr. William C. Claycomb [51] in Louisiana State University Medical Center, New Orleans, LA. The cells were maintained in Claycomb basal medium (Sigma) supplemented with 10% fetal bovine serum, 0.1 mM norepinephrine and 2 mM L-glutamine. HL-1 cells were treated with adriamycin at 0 μmol/L, 0.25 μmol/L, 0.50 μmol/L, or 1.00 μmol/L for 48 hrs in complete growth medium, or add ANGPTL8 gene plasmids transfection with lipofectamin2000, or 2 pmol/L ANGPTL8 peptide (Phoenix Pharmaceuticals, Burlingame, CA, USA) to a dish and were subjected to immunofluorescent staining.

### ELISA for detecting plasma ANGPTL8

Fasting venous blood was collected in EDTA tubes from the tail vein, and plasma lipids were measured using standard enzymatic assays. Human ANGTPL8 was measured using ELISA (Phoenix Pharmaceuticals, Burlingame, CA, USA).

### RNA isolation and quantitative RT–PCR analysis

Total RNA was isolated from 100 mg of heart using the RNeasy mini kit (Qiagen). Real-time quantitative RT-PCR (qRT-PCR) analysis was performed on an ABI 7700 Sequence Detector (Applied Biosystems, Grand Island, NY, USA) using SYBR Green (RT2 SYBR Green qPCR Kit; Qiagen, Boston, MA, USA). Data were normalized to the expression of housekeeping genes (as an endogenous control). Changes in gene expression were normalized to control liver and cardiac muscle samples.

### Western blotting

Total protein extracts from heart tissue were evaluated with a Cytoplasmic and Nuclei Extraction Kit (Thermo Scientific, Rockford, IL, USA). Protein concentrations were determined using the BCA-200 Protein Assay kit (Pierce, Grand Island, NY, USA); equal amounts of protein were separated by SDS-PAGE to nitrocellulose membranes and incubated with primary antibodies anti ANGPTL8 (1:1,000 dilutions), anti-PirB(1:2000 dilution), anti ISL-1 (1:1000 dilution), anti PHH3 (1:2000 dilution) and anti-actin (1: 2,000 dilutions) and anti-Lamin (1:2000 dilution). Horseradish peroxidase secondary antibodies were used, and chemiluminescence was determined using the SuperSignal West Dura detection system (Pierce); Cytoplasmic marker (actin) and nuclei marker (lamin) were used to confirm equal loading. All Western blots were performed in duplicate.

### Echocardiography

Echocardiographic measurements of LV mass, fractional shortening and LV posterior wall thickness were made from digital images acquired with a 12 MHz broadband transducer (S12 probe, Philips Ultrasound, Bothell, WA) in M-mode under 2D echo short axis view. LV mass=1.05[LVIDd +IVS+LVIPW]^3^-[LVIDd]^3^. Fractional shortening was evaluated from the following formula: FS=(LVIDd-LVIDs)/LVIDd X 100.

### Data analysis

Data were analysed using Statview software (SAS, Cary, NC, USA). The values are presented as mean ± SEM. Differences were analyzed by repeated measures ANOVA with Fisher's post hoc test and were considered significant at p<0.05.

### Supplementary material

Supplementary material is available at Oncotarget online.

## SUPPLEMENTARY MATERIAL


