# Supplementary material for: ANGPTL8 reverses established adriamycin cardiomyopathy by stimulating adult cardiac progenitor cells
Source: Oncotarget. 2016 Nov 3;7(49):80391–403. doi: 10.18632/oncotarget.13061 (PMC5348328; doi:10.18632/oncotarget.13061)
Supplement: Supplementary file 1 [file oncotarget-07-80391-s001.pdf]

ANGPTL8 reverses established adriamycin cardiomyopathy by stimulating adult cardiac progenitor cells

Supplementary Material

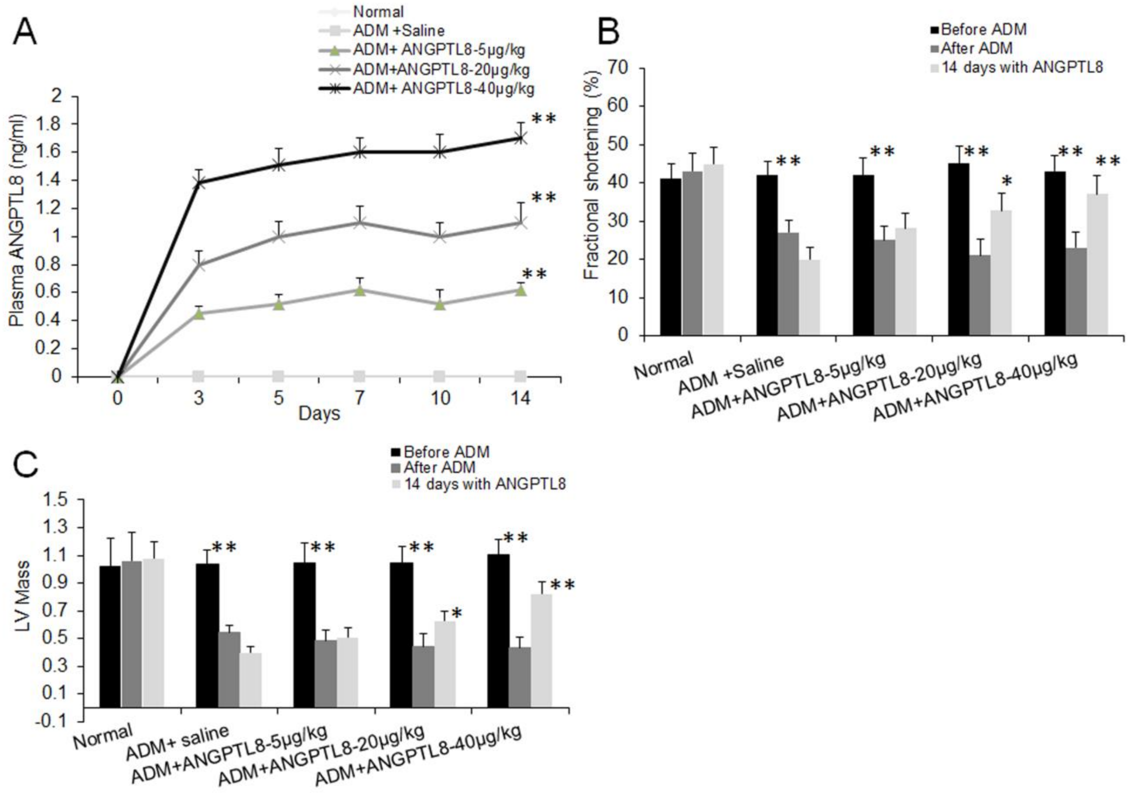

Supplemental Figure 1. **ANGPTL8 protein intramuscular injection reversed established ADM cardiomyopathy. (A)** a graphic of fasting plasma human *ANGPTL8* levels. In normal and ADM + saline inject, human *ANGPTL8* was not detectable. Values are presented as mean ± SEM. *n* =3 per group; **P<0.001 *vs* control groups. ANGPTL8 protein injection at doses of 5μg-20μg-40μg/kg/day for 14 days; **(B)** a graphic of fractional shortening, **(C)** a graphic of LV mass, Values are presented as mean±SEM. *n* = 3 per group; **P<0.001 and *<0.05 *vs* after ADM.

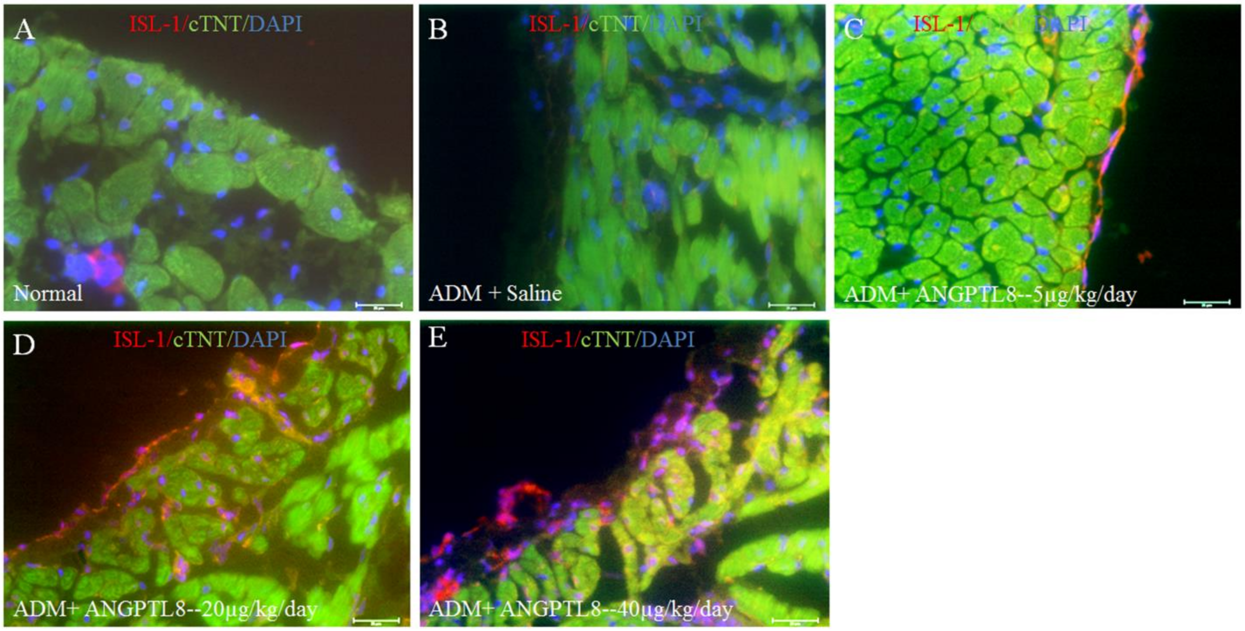

Supplemental Figure 2. ANGPTL8 protein therapy inducing the activation of ISL-1 (an early cardiac muscle differentiation marker) in epicardium and sub-epicardial layer. (A) normal rat heart, **(B)** ADM + Saline injection, **(C)** ADM plus ANGTPL8 protein 5 µg/kg/day for 14 days, **(D)** ADM plus ANGTPL8 protein 20 µg/kg/day for 14 days, (E) ADM plus ANGTPL8 protein 40 µg/kg/day for 14 days. Scale bar is 25 µm.

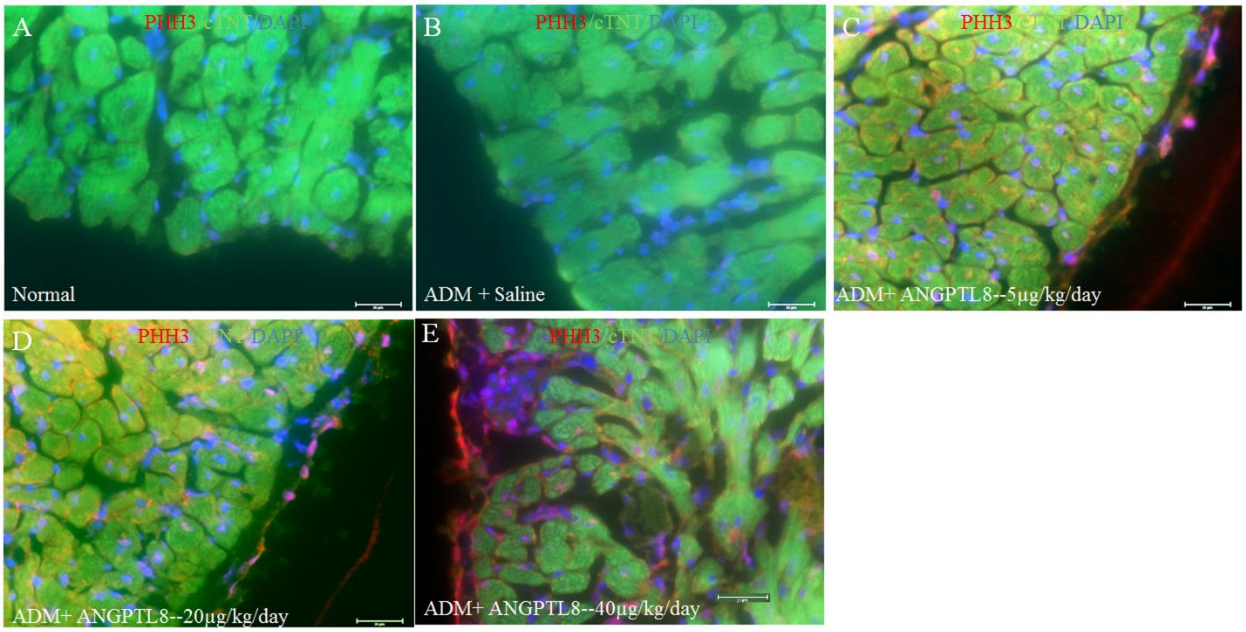

Supplemental Figure 3. **Phospho-histone H3 (PHH3) staining shown some epicardial cells and sub-epicardial cardiac muscle cells are in proliferation after ANGPTL8 protein therapy. (A)** normal rat heart, **(B)** ADM + Saline injection, **(C)** ADM plus ANGTPL8 protein 5 μg/kg/day for 14 days, **(D)** ADM plus ANGTPL8 protein 20 μg/kg/day for 14 days, **(E)** ADM plus ANGTPL8 protein 40 μg/kg/day for 14 days. Scale bar is 25 μm.

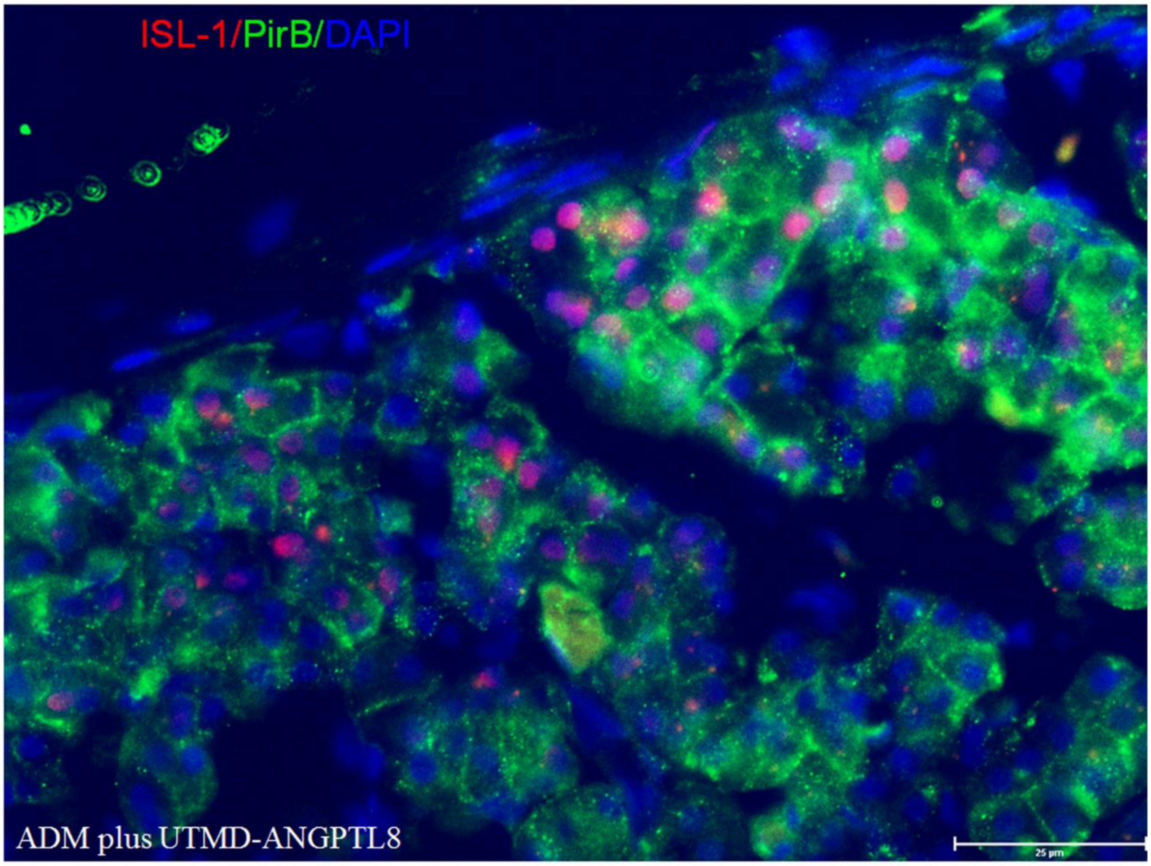

Supplemental Figure 4. **PirB distributed in membrane of ISL-1 positive cardiac progenitor cells.** Scale bar is 25 μm.

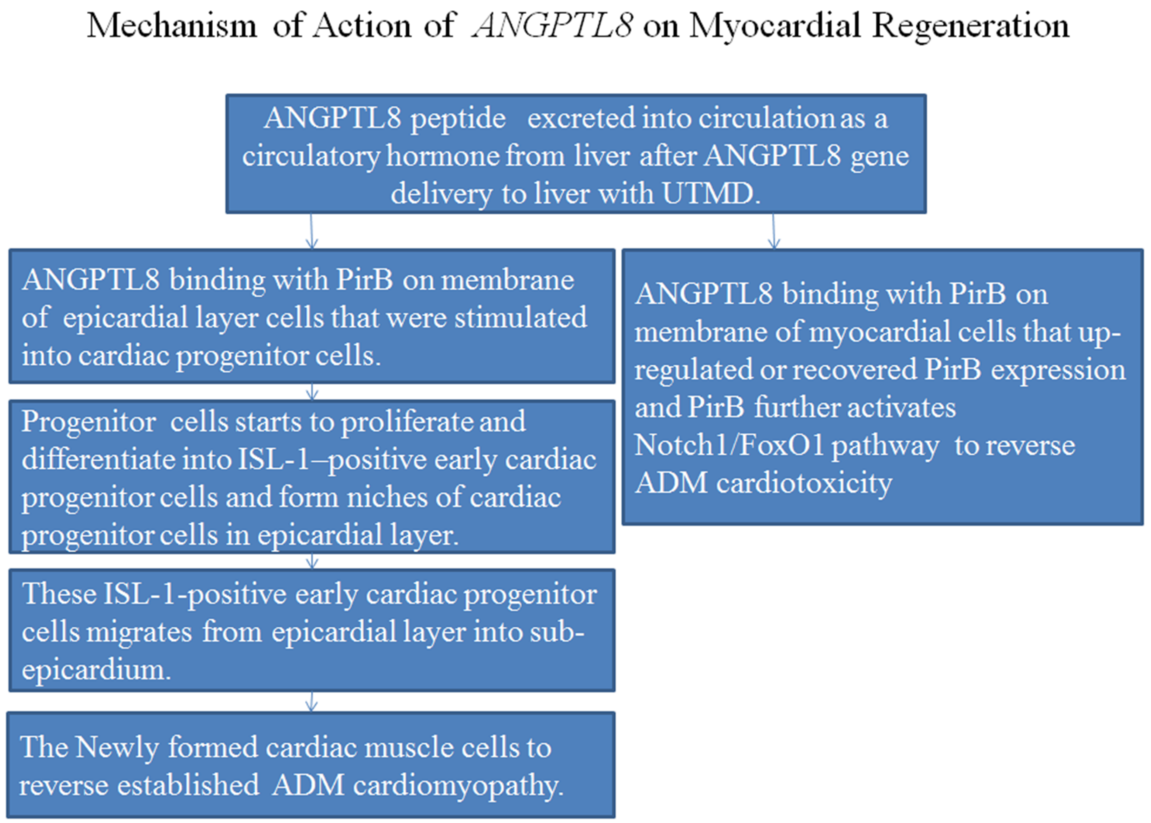

Supplemental Figure 5. Schematic indicating mechanism of action of ANGPTL8 on myocardial regeneration.
